# Anti-Inflammatory Effects of Rosiglitazone in Obesity-Impaired Wound Healing Depend on Adipocyte Differentiation

**DOI:** 10.1371/journal.pone.0168562

**Published:** 2016-12-19

**Authors:** Anna Siebert, Itamar Goren, Josef Pfeilschifter, Stefan Frank

**Affiliations:** Pharmazentrum Frankfurt/ZAFES, Klinikum der Johann Wolfgang Goethe-Universität, Theodor-Stern-Kai 7, Frankfurt am Main, Germany; University of Cambridge, UNITED KINGDOM

## Abstract

Combined diabetes-obesity syndromes severely impair regeneration of acute skin wounds in mouse models. This study assessed the contribution of subcutaneous adipose tissue to exacerbated wound inflammatory conditions. Genetically obese (*ob/ob*) mice showed an increased expression of positive transcriptional effectors of adipocyte differentiation such as Krüppel-like factor (KLF)-5 and peroxisome proliferator-activated receptor (PPAR)-γ and an associated expression of leptin and fatty acid-binding protein (FABP)-4, but also CXCL2 in isolated subcutaneous fat. This observation in obese mice is in keeping with differentially elevated levels of KLF-5, PPAR-γ, leptin, FABP-4 and CXCL2 in *in vitro*-differentiated 3T3-L1 adipocytes. Notably, CXCL2 expression restrictively appeared upon cytokine (IL-1β/TNF-α) stimulation only in mature, but not immature 3T3-L1 adipocytes. Of importance, the critical regulator of adipocyte maturation, PPAR-γ, was merely expressed in the final phase of *in-vitro* induced adipocyte differentiation from 3T3-L1 pre-adipocytes. Consistently, the PPAR-γ agonist rosiglitazone suppressed cytokine-induced CXCL2 release from mature adipocytes, but not from early 3T3-L1 adipocyte stages. The inhibitory effect of PPAR-γ activation on CXCL2 release appeared to be a general anti-inflammatory effect in mature adipocytes, as cytokine-induced cyclooxygenase (Cox)-2 was simultaneously repressed by rosiglitazone. In accordance with these findings, oral administration of rosiglitazone to wounded obese mice significantly changed subcutaneous adipocyte morphology, reduced wound CXCL2 and Cox-2 expression and improved tissue regeneration. Thus, our data suggest that PPAR-γ might provide a target to suppress inflammatory signals from mature adipocytes, which add to the prolonged wound inflammation observed in diabetes-obesity conditions.

## Introduction

As anticipated more than a decade ago [1], diabetes mellitus (type 2) has now developed into a full global epidemic [2]. Population ageing, urbanization and sedentary lifestyle [1] will particularly contribute to the projection of 439 million concerned people by 2030 [3]. The point at issue is that the observed global expansion of diabetes mellitus is connected to an increasing incidence of overweight and obesity, with an estimated 57% of the world-wide adult population that will be afflicted in 2030 [4]. In particular, diabetes-associated skin ulcerations often constitute a severe complication of the disease and thus a still unsolved medical condition associated with a significant mortality [5,6]. More important, the overall prognosis of diabetic ulcers is poor, and concerned patients suffer from low 3-year survival rates between 50% and 59% [7,8].

Starting in the early 1970s, the first cutaneous wound healing models were established in guinea pigs to determine basic cellular and molecular repair processes such as the role of neutrophils [9], macrophages [10] or complement [11]. However, the necessity to unravel basic cellular and molecular mechanisms that might represent the basis of diabetes-impaired wound healing conditions led to a comprehensive use of two obesity-linked mouse models of type 2 diabetes in this field of research: the diabetes/diabetes (*db/db*) and obese/obese (*ob/ob*) mouse strains, which both combine an obese and diabetic phenotype [12–17]. The obesity-linked diabetic phenotype of both mouse strains is caused by a defect in leptin signaling constituting from mutations in leptin (*ob/ob* mice) [18] or the leptin receptor (*db/db* mice) [19], respectively. In addition, leptin represents a pivotal regulator of inflammatory processes, although its actions appeared to be tissue-specific. Whereas leptin supplementation markedly attenuated the inflammatory response in skin wounds in leptin-deficient *ob/ob* mice by reduction of wound macrophage inflammatory potencies [20], leptin replacement increased the severity of intestinal inflammation by augmentation of neutrophil influx and cytokine production in ob/ob mice [21]. Thus, leptin also represents an endocrine signaling molecule connected to the immune system, but leptin’s regulatory potencies might be fundamentally different for different organ systems.

Both *ob/ob* and *db/db* mouse strains are characterized by formation of severely impaired wounds upon skin injury. In the animals, the disturbed processes of keratinocyte proliferation, re-epithelialization and granulation tissue formation [14–16] under conditions of absent or inactive growth factors [22–24] and an exacerbated inflammation [17,20,25] integrate into severe wound tissue defects. Here it is tempting to recognize that both *db/db* and *ob/ob* mouse models have been in experimental use because of their diabetic phenotype [26]. Actually, the diabetic phenotype of the animals is functionally connected to the presence of an extensive adipose tissue mass [26,27]. This notion means, most notably, that these two widely used experimental mouse models of diabetes mellitus are not based, as one would expect, on a genetic defect concerning the insulin/insulin receptor signaling cascades. Instead, the diabetic phenotype of the animals originates from a functional defect in leptin signaling [26], an adipocyte-derived cytokine that normally serves the communication between adipose tissue and the hypothalamus in the CNS [28]. Thus, the observed insulin resistance in these experimental models of diabetes-impaired wound healing actually reflects a functional consequence of a hypertrophic and hyperplastic adipose tissue.

In view of this notion, it is remarkable that the role of the massive subcutaneous adipose tissue, that also directly underlines acute wounds areas, has not attracted interest in terms to analyze the contribution of fat cells to impaired wound conditions. Adipose tissue comprises a variety of cell types such as endothelial cells, fibroblasts, pericytes, macrophages and T-cells with the mature adipocyte as the main cell type [29]. *In vitro* models of pluripotent fibroblasts or fibroblast-like preadipocytes [30] identified activator protein (AP)-1 [31], Krüppel-like factor (KLF)-5 [32], CAAT/enhancer-binding protein (C/EBP) [33] or peroxisome proliferator-activated receptor (PPAR)-γ [34,35] as key transcription factors of adipogenesis. In particular, KLF5 expression during early stages represents a prerequisite for adipocyte differentiation [32] and PPARγ is now established as the master regulator of adipocyte differentiation and sufficient to drive adipocyte development [34,35]. As a direct consequence, PPARγ knock out mice fail to develop a functional adipose tissue [36,37] and are protected from obesity-induced insulin resistance [38]. There are two known isoforms to PPARγ, PPARγ1 and PPARγ2. The latter isoform includes an additional 30 amino acids at its N-terminus [39]. PPARγ1 is expressed in many tissues including leukocytes, particularly macrophages, whereas PPARγ2 is normally restricted to adipose tissue, but can be induced elsewhere [39–42].

Notably, obese *db/db* and *ob/ob* mice suffer from exacerbated wound inflammatory conditions [17,20,25]. Large adipocytes drive an elevated release of the pro-inflammatory factors IL-6, CXCL2 or MCP-1, or CrP [43], however, a direct contribution of adipocyte-derived inflammatory signals to wound inflammation has been insufficiently addressed yet. To this end, we assessed the inflammatory response of differentiating adipocytes during PPARγ-dependent adipogenesis *in vitro*. This study shows that PPARγ2, which is expressed in mature adipocytes [34,35], can be specifically targeted by thiazolidinediones [44] to reduce CXCL2 and cyclooxygenase (Cox)-2 expression in cultured mature adipocytes and in inflamed wound tissue of obese mice.

## Materials and Methods

### Animals

Female C57Bl/6J (wild-type) and C57Bl/6J-*ob/ob* mice were obtained from Charles River (Sulzfeld, Germany). At the age of 12 weeks, mice were caged individually using cages with an enriched environment. Prior to wounding, mice were randomly assigned to different experimental groups, monitored for body weight, and wounded as described below. The animal experiments were performed according to the guidelines and approval of the local Ethics Animal Review Board (Regierungspräsidium Darmstadt, D-64278 Darmstadt, Germany). The approval number to this project was V54-19c20/15 –F143/10).

### Treatment of mice

Rosiglitazone (Avandia, GlaxoSmithKline) was emulsified (2 mg/ml) in 0.5% Tylose solution (Sigma, Taufkirchen, Germany) and administered orally once a day by gastrogavage (0.5 mg/kg/day) during wound healing. 0.5% Tylose solution alone served as mock-treated control. Treatment started two days prior to wounding.

### Wounding of mice

Wounding of mice was performed as described previously [45,46]. Briefly, mice were anesthetized using Ketamin (80 mg/kg) for analgesia and Isofluran (4% vol). Subsequently, six full-thickness wounds (5 mm in diameter, 3–4 mm apart) were made on the backs of the mice by excising the skin and the underlying *panniculus carnosus*. The wounds were allowed to form a scab. Mice were sacrificed by cervical dislocation and subsequent bleeding. An area of 7–8 mm in diameter, which included the granulation tissue and the complete epithelial margins, was excised at the indicated time points for analysis. Back skin from non-wounded mice served as a control. For every experimental time point showing data from wounded animals, we have analyzed 12 individually isolated wound tissue areas (n = 12) isolated from 4 individual animals (n = 4) for RNA analysis. For protein analysis, 8 individual wounds (n = 8) from 4 individual mice (n = 4) were used. For RNA analysis of subcutaneous fat, we isolated subcutaneous fat samples from four non-wounded individual mice (n = 4).

### Isolation of adipose tissue

Mice were sacrificed by cervical dislocation and subsequent bleeding. Underlying hypertrophic white adipose tissue was isolated from mice upon detachment of complete skin tissue (including the panniculus carnosus). We used this adipose tissue layer, as the wounding procedure completely removes the panniculus carnosus (see above) at the wound site and exposes exactly this adipose tissue compartment beneath the wound site. The small fat compartment between epidermis/dermis and panniculus carnosus is removed during wounding and does not represent the fat tissue of the wound bed. The analyzed subcutaneous adipose tissue compartment is highlighted in S1 Fig. Inguinal subcutaneous fat was not used for analysis.

### Oral glucose tolerance test (OGT)

ob/ob mice were analyzed after 15 days of vehicle (mock) or rosiglitazone treatment. Mice were starved for 16 h and subsequently administered glucose orally (1.5 g/kg body weight) by gastrogavage. Blood glucose levels were determined before and 30, 60, 90 and 120 min after glucose application.

### Cell culture

Dulbeccco’s modified Eagle’s medium (DMEM)-high glucose (4500 mg/ml), DMEM/F12 (1:1), Penicillin/Streptomycin (Pen/Strep) and 0.05% Trypsin-EDTA were purchased from Life Technologies (Thermo Fisher Scientific Darmstadt, Germany). Fetal bovine serum (FBS) was obtained from Biochrom (Berlin, Germany). NIH/3T3 cells (ATCC-CRL-1658), the pre-adipocyte cell-line NIH/3T3-L1 (ATCC-CL-173) and new-born calf bovine serum (NCS) (ATCC-30-2030) were purchased from ATCC (LGC Wesel, Germany). All tissues and cells were maintained in supplemented medium containing 10% (v/v) heat inactivated NCS or FCS and 100 U/ml Pen/Strep in a 37°C humidified environment containing 5% CO_2_. NIH 3T3-L1 pre-adipocytes were maintained in DMEM supplemented with 10% NCS and 100 U/ml Pen/Strep (basal medium I, BMI) to reach 80% confluency. At a cell density of 5 x 10^4^ cells/cm^2^, cells were trypsinized and seeded at 6.9 x 10^4^ cells/cm^2^ in 35 mm dishes. Cells were then allowed to attach for 48 hours. Differentiation of the pre-adipocytes was subsequently induced by a medium change from BMI to differentiation medium I (DMI). DMI contained 10% FCS, 100 U/ml Pen/Strep, insulin (1μg/ml), 0.5 mM 3-isobutyl-1-methylxanthine (IBMX), 0.25 μM dexamethasone and 2 μM rosiglitazone in DMEM for additional 48 hours. DMI was replaced by differentiation medium II (DMII) containing 10% FCS, 100 U/ml Pen/Strep and insulin (1μg/ml) in DMEM. Over a period of 10 days, DMII was replaced every other day to allow final differentiation of the cells. To control final adipocyte differentiation, fat droplet accumulation in the cells was assessed using Oil Red O staining [47].

### RNA isolation

RNA isolation was performed as described previously [48]. Every wound time point depicts a total of 12 wounds (n = 12) isolated from four individual mice (n = 4) for analysis. Total RNA from subcutaneous fat tissue was isolated from four individual mice (n = 4) for analysis. For cell culture experiments, total RNA was isolated from three independent cell culture experiments (n = 3).

### Quantitative real-time polymerase chain reaction (qRT-PCR)

qRT-PCR was performed to assess the expression of CXCL2, CCL5, leptin, fatty acid binding protein (FABP)-4, krüppel-like factor (KLF)-5, peroxisome proliferator-activated receptor (PPAR)-γ1, PPAR-γ2, CCAAT/enhancer-binding protein (C/EBP)α, and cyclooxygenase (Cox)-2. TaqMan® FastAdvanced Master mix (cat. No. 4444557) and the pre-designed qRT-PCR assays were purchased at Applied Biosystems (Darmstadt, Germany): mouse CXCL2 (Mm00436450_m1), mouse CCL5 (Mm01302427_m1), mouse leptin (Mm00434759_m1), mouse FABP4 (Mm00445878_m1), mouse KLF-5 (Mm00456521_m1), mouse PPARγ2 (Mm00440940_m1), mouse C/EBPα (Mm00514283_m1), or mouse Cox-2 (Mm00478374_m1). qRT-PCR was performed on 7500 Fast real-time PCR system (Applied Biosystems) as follows: 95°C (20 s), 40 cycles: 95°C (3 s) and 60°C (30 s). Relative changes in the respective mRNA expression were normalized to murine GAPDH (4352339E, VIC) or to murine eukaryotic translation elongation factor 2 (EEF2) (Mm01171435_gH). To evaluate the expression of PPARγ1, the following forward primer (5’-GAA TAC CAA AGT GCG ATC AAA GT-3’) and reverse primer (5’-GGA AAA AAC CCT TGC ATC CT-3’) and the Dynamo-Color-Flash SYBR Green qPCR Kit (F-415L; Biozyme Scientific (Wien, Austria) were used. Detection of the quenched probe, calculation of cycle threshold (Ct) values, and data analyses were performed by the Sequence Detector software and evaluated according to the 2^-ΔCT^ method.

### Preparation of protein lysates and Western blot analysis

Tissue biopsies from non-wounded skin, wounds, subcutaneous fat, or cell culture samples were homogenized in lysis buffer (1% Triton X-100, 20 mM Tris/HCl pH 8.0, 137mM NaCl, 10% glycerol, 1 mM DTT, 5 mM EDTA, 10 mM NaF, 2 mM Na_3_VaO_4_, 1 mM PMSF, 5 ng/ml aprotinin, 5 ng/ml leupeptin 50 and nM Okadaic acid). Extracts were cleared by centrifugation. Protein concentrations were determined using the BCA Protein Assay Kit (Pierce Inc., Rockford, IL, USA). Twenty five to fifty micrograms of total protein lysates were separated using SDS gel electrophoresis. After transfer to a nitrocellulose membrane and blocking in 10 mM Tris-HCl, 140mM NaCl and 0.05% Tween 20 (pH 8.0) containing 2.5% nonfat milk, the membranes were incubated with primary antibodies for 16 to 18 hours at 4°C. A secondary antibody against goat, rabbit or mouse IgG coupled to horseradish peroxidase (Bio-Rad, Munich, Germany), and the enhanced chemiluminescence (ECL) detection system (Amersham, Freiburg, Germany) was used to visualize the proteins.

### Enzyme-linked immunosorbent assay (ELISA)

Cell culture supernatants were collected and cleared by brief centrifugation (300 x g, 5 min at 4°C). Tissue biopsies were prepared as described. Fifty μl of culture supernatants or 12.5 to 50 μg of total protein from tissue biopsies were used for analysis. Quantification of murine CXCL2 and leptin was conducted using murine CXCL2 DuoSet and leptin quantikine ELISA kits (R&D Systems, Wiesbaden, Germany). Murine CCL5 was quantified by use of Elisa Development Kit (PeproTech, Hamburg, Germany) according to the instructions of the manufacturer.

### Heidenhains’s AZAN trichrome staining

Wounded mice were euthanized at day 13 after injury. Biopsies from skin wounds were isolated from the back, fixed in formalin and embedded in paraffin. Four-micrometer sections were rehydrated and stained using 0.1% (w/v) Azocarmine G (Sigma, Taufkirchen, Germany) in 1% (v/v) acetic acid for 7 min. Sections were washed with 1% (v/v) acetic acid followed by oxidation in 5% (w/v) wolframato phosphoric acid (Sigma) for 20 minutes and rinsed in water. Sections were then counterstained using 1.5% (w/v) Aniline Blue/Orange G (Sigma) for 4 minutes, washed in water, dehydrated and mounted using Entellan^®^ water-free mounting medium (Merck, Darmstadt, Germany).

### Generation expression of recombinant C/EBPα (rC/EBPα), rKLF5 and rPPARγ proteins

Full length mouse C/EBPα, PPARγ1, PPARγ2 and KLF5 open reading frames (ORFs) were amplified from day 3 wound-RNA using reverse transcriptase and polymerase chain reaction according to standard DNA cloning protocols. PCR was performed on a T3000-Thermocycler (Biometra, Göttingen, Germany). Primers were 5’-GCA TGG TAC CAC CAT GGA GTC GGC CGA CTT CT-3’ (forward) and 5’-GCT AGG TGA CCC GCG CAG TTG CCC ATG GCC T-3’ (reverse) for C/EBPα; 5’-GCT AGC TAG CCA CCA TGG TTG ACA CAG AGA TGC CA-3’ (forward) and 5’-CGA TGG TGA CCA TAC AAG TCC TTG TAG ATC TCC T-3’ (reverse) for PPARγ1; 5’-GCT AGC TAG CCA CCA TGG GTG AAA CTC TGG GAG-3’ (forward) and 5’-CGA TGG TGA CCA TAC AAG TCC TTG TAG ATC TCC T-3’ (reverse) for PPARγ2. CEBPα, PPARγ1 and PPARγ2 amplicons were digested and cloned into pcDNA3-1-myc-HisC (Invitrogen, Karlsruhe, Germany) *via* Kpn/BstEII restriction sites to generate the CEBPα-, PPARγ1- and PPARγ2- Myc-6Histidine-tagged expression plasmids: pcCEBPα-, pcPPARγ1- and pcPPARγ2-Myc6HisC, respectively. For murine KLF5, the following primers 5’-GCATGCTAGCCACCATGCCCACGCGGGTGCTGA-3’ (forward) and 5’- CGATGGATCCGTTCTGGTGGCGCTTCATGT-3’ (reverse) were used. KLF5 amplicons were digested and cloned into of pCMV Akt1 FLAG-N3 [49] via NheI/BamHI restriction sites to generate the KLF5-Flag-tagged expression plasmid pCMVKLF5.

### Reagents

Phenylmethylsulfonyl fluoride (PMSF), dithiothreitol (DTT), aprotinin, NaF and Na_3_VaO_4_ and ethylenediaminetetraacetic acid (EDTA), dexamethasone, human insulin, 3-isobutyl-1-methylxanthine (IBMX) and Oil-Red were from Sigma (Taufkirchen, Germany). Leupeptin and ocadaic acid were from BioTrend (Cologne, Germany). Rosiglitazone was obtained from Cayman (Biozol Eching, Germany). Deoxynucleoside triphosphate and Random hexamers were obtained from Roche Diagnostics (Mannheim, Germany). DNA restriction endonucleases were obtained from New England Biolabs (Frankfurt am Main, Germany). KAPAHiFi polymerase was purchased from Peqlab Biotechnologie GmbH (Erlangen, Germany). Purified recombinant mouse cytokines and were acquired from PeproTech Inc. (Hamburg, Germany).

### Antibodies

Rabbit anti GAPDH (2275- PC- 100) (Trevinogen; Gaithersburg, Maryland, USA); rabbit anti-mouse CEBPα, (sc-61); goat anti-human PPARγ2, (sc-22022); mouse monoclonal (E-8) anti-human PPARγ1 (sc-7273) (Santa- Cruz, Heidelberg, Germany); rabbit monoclonal anti-human phospho-p44/42 MAPK (CST 4376); rabbit anti-human p44/42 MAPK (CST-9102); rabbit monoclonal (81E11) anti-human phospho-SAPK/JNK (CST 4668); rabbit anti-human SAPK/JNK (CST 9252); rabbit monoclonal (44D4) anti-human IκB (CST-4812),), rabbit monoclonal (93H1) anti-human phospho-p65 (Ser536) (CST 3033), rabbit monoclonal (C22B4) anti-human NF-κB p65 (CST 4764) (Cell Signaling, Frankfurt, Germany); rabbit anti-mouse KLF5 (07–1580, Millipore Merck, Darmstadt, Germany); rabbit anti-mouse Cox2 (Cayman 160126; Tallinn, Estonia) and mouse monoclonal (AC-15) anti-β- Actin, (A5441, Sigma).

### Statistical analysis

Data are shown as means ± SD. Data analysis was carried out using the unpaired Student's *t* test with raw data and graph pad prism software, version 5.02.

## Results

### Expanded white adipose tissue exhibits an altered gene expression

**Fig 1:**
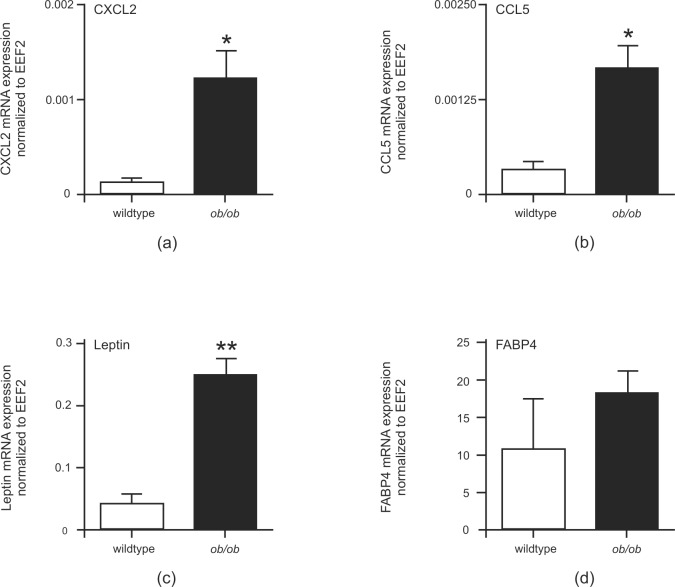
Altered gene expression in subcutaneous fat tissue of obese mice. qRT-PCR quantification of CXCL2 (a), CCL5 (b), leptin (c) and FABP4 (d) mRNA expression in subcutaneous fat tissue of wildtype and obese (*ob/ob*) mice as indicated. **, p < 0.01; *, p < 0.05 (Student’s unpaired *t* test) as compared to wildtype mice. Bars indicate the mean ± S.D. obtained from fat tissue isolated from four individual animals (n = 4).

**Fig 2:**
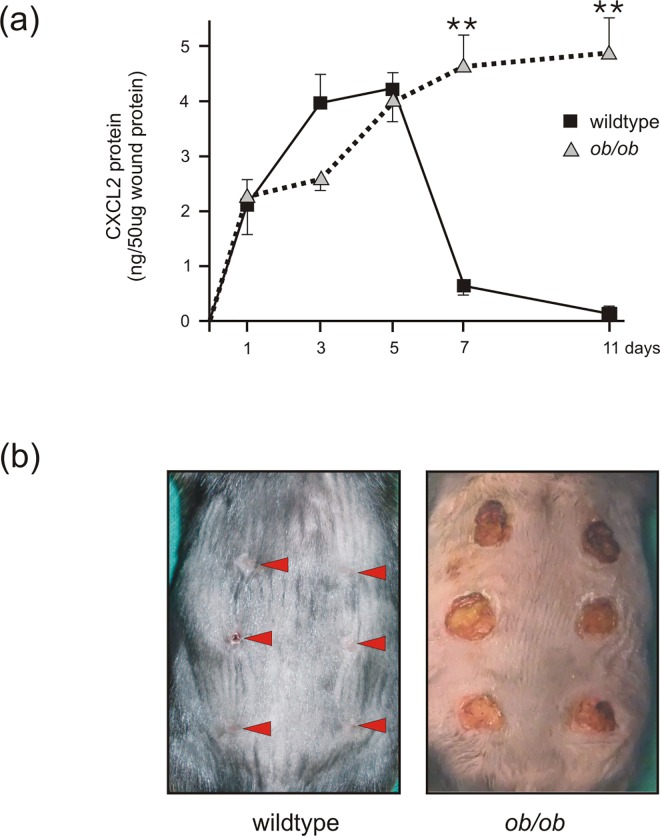
Elevated levels of CXCL2 protein in wounds of wild-type and obese mice. (a) Analysis of CXCL2 protein expression by ELISA from skin and wound tissue at different time points in wild-type and obese (*ob/ob*) mice as indicated. **, p < 0.01; (Student’s unpaired *t* test) compared to wildtype mice. Bars indicate the mean ± S.D. obtained from wounds (n = 8) isolated from four individual animals (n = 4). (b) Back wound phenotype of wildtype and ob/ob mice at day 11 upon wounding as indicated. Arrows highlight the wound areas in the wildtype mouse.

It has long been known that expression of a large number of transcripts correlated significantly with body mass [50]. Therefore, it was worth to assess a possible role of adipose tissue alterations in the context of diabetes, obesity and wound healing. We isolated subcutaneous white fat tissue from healthy wildtype and diabetic and obese *ob/ob*-mice [26]. Here, Fig 1A and 1B show that the hypertrophic white fat cell mass in *ob/ob*-mice was characterized by elevated expression levels of the representative leukocyte-recruiting chemokines CXCL2 or CCL5. In addition, the mRNA encoding fat cell-derived leptin [18] was increased in obese mice (Fig 1C). Also FABP4 [45] mRNA showed a tendency to rise in obese mice (Fig 1D). In view of the elevated levels of CXCL2 (Fig 1) in subcutaneous fat tissue, it was interesting to observe largely elevated levels of CXCL2 protein in late wound tissues of obese *ob/ob* mice (Fig 2A). As also shown previously for *db/db* mice [17], increased levels of CXCL2 protein in wound tissue of *ob/ob* mice were associated with a delayed wound closure as compared to wildtype mice (Fig 2B). Whereas lean wildtype mice showed a transient and restricted expression of CXCL2 protein during the acute phase or repair, it was tempting to further assess a potential contribution of adipose tissue to the sustained dysregulation of inflammatory mediators such as CXCL2 in obesity-impaired wound tissue (Fig 2).

### Differentiation renders adipocytes susceptible to pro-inflammatory signals

**Fig 3:**
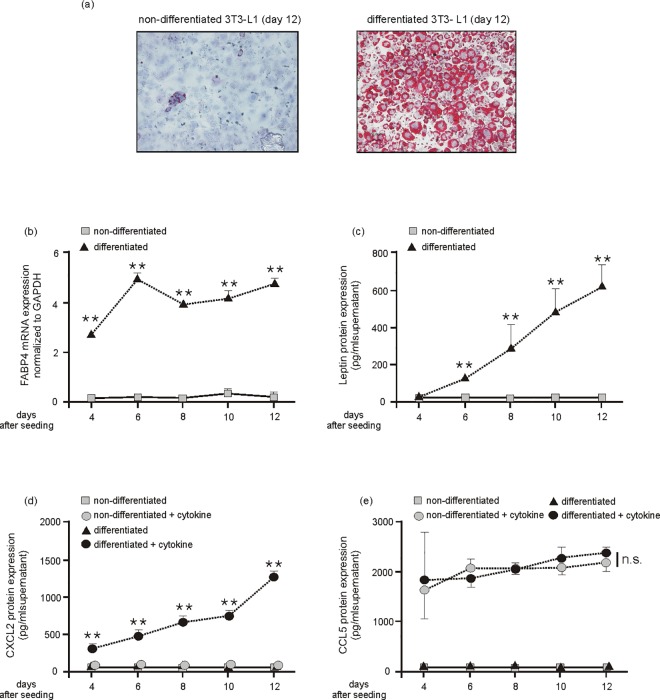
Cytokine-induced CXCL2 is dependent on adipocyte differentiation. Cultured 3T3-L1 pre-adipocytes were differentiated in DMI and DMII for 12 days. Final differentiation was assessed by Red Oil staining (a). Non-differentiating and differentiating 3T3-L1 adipocytes were assessed by qRT-PCR analysis for FABP4 mRNA expression (b) and leptin protein release into culture media (c) at the indicated time points. **, p < 0.01; (Student’s unpaired *t* test) compared to non-differentiating cells. Bars indicate the mean ± S.D. obtained from four independent experiments (n = 4). Non-differentiating and differentiating 3T3-L1 adipocytes were stimulated with fresh medium in the absence or presence of cytokines (25 ng/ml IL-1β, 50 ng/ml TNFα) for 8h at the indicated time points. Conditioned media from the cells were analyzed by ELISA for CXCL2 (d) and CCL5 (e) protein expression. **, p < 0.01; n.s., not significant (Student’s unpaired *t* test) compared to cytokine-treated non-differentiating cells. Bars indicate the mean ± S.D. obtained from four independent experiments (n = 4).

The 3T3-L1 cell line is an extensively characterized adipocyte cell line [51]. Thus, we differentiated 3T3-L1 cells in culture (Fig 3) to assess those proteins that have been observed to be upregulated in the expanded adipose tissue of obese mice (Fig 1). First we confirmed the 3T3-L1 differentiation protocol. 3T3-L1 adipocytes were stained using OilRed to verify the formation of intracellular oil droplets as a marker of final differentiation (Fig 3A). In accordance with the increased expression in adipose tissue of obese mice (Fig 1), we found markedly elevated levels of the adipocyte marker transcripts FABP4 (Fig 3B) and leptin (Fig 3C) to be associated with the gradually progressing differentiation process in 3T3-L1 cells. By contrast, expression of the pro-inflammatory mediators CXCL2 and CCL5 were not induced during the adipocyte differentiation process (Fig 3D and 3E). However, it is important to note that the differentiation process towards mature adipocytes appeared to be a prerequisite for the robust induction of CXCL2 (Fig 3D), but remarkably not of CCL5 (Fig 3E) upon cytokine stimulation of the cells. Here, it is important to note that the pro-inflammatory cytokines (IL-1β, TNFα) had not been added during the differentiation process of the cells to avoid cytotoxic effects to pre-adipocytes or inhibition of adipocyte maturation. The cytokines were therefore added for only 8 h not until the cells had reached the given experimental differentiation time points (day 4, 6, 8, 10, 12). Interestingly, non-differentiated 3T3-L1 cells did not express CXCL2 at all.

**Fig 4:**
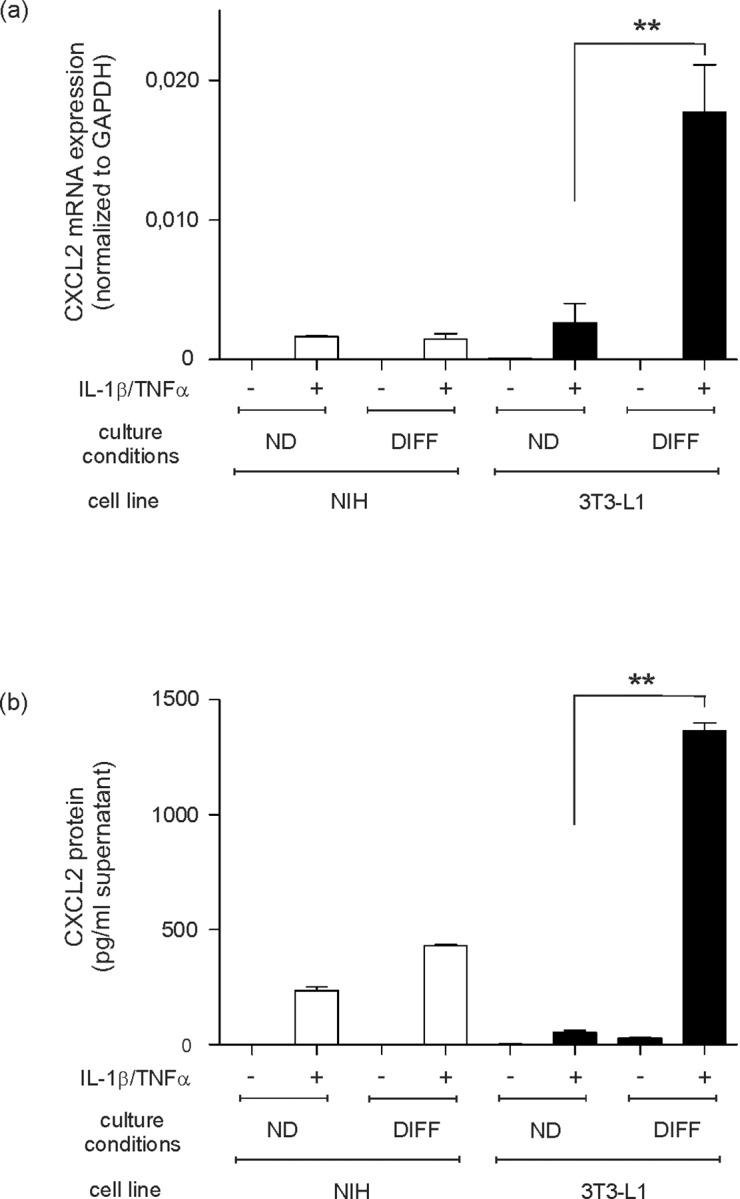
Differentiation-dependent expression and release of CXCL2 in 3T3-L1 cells. NIH 3T3 fibroblasts and 3T3-L1 pre-adipocytes were cultured in differentiation medium (*DIFF*) or remained undifferentiated (*ND*) in control medium (DMEM) for 12 days. At day 12, differentiated (*DIFF*) and non-differentiated (*ND*) 3T3-L1 cells were stimulated with cytokines (25 ng/ml IL-1β, 50 ng/ml TNFα) as indicated. NIH 3T3 fibroblasts served as a control. CXCL2 mRNA expression was analyzed by qRT-PCR (a). CXCL2 protein release into cell culture supernatants was determined by ELISA (b). **, p < 0.01; (Student’s unpaired *t* test) compared to non-differentiated cells. Bars indicate the mean ± S.D. obtained from four independent experiments (n = 4).

It is of importance that 3T3-L1 cells possess the capability to synthesize high amounts of collagen prior to its adipose conversion. The cells might therefore be considered as a fibroblast line with an additional form of specialization [51]. Therefore, we also treated cultured NIH 3T3 fibroblasts using cytokines. We did so to determine whether the observed expression of CXCL2 and CCL5 (Fig 3D and 3E) resembles a general capability of fibroblastic cells or a particular feature of maturating 3T3-L1 cells. Remarkably, NIH 3T3 fibroblasts were not capable to produce marked amounts of CXCL2 mRNA and protein upon TNFα/IL1β stimulation in the absence or presence of adipocyte differentiation supplement (Fig 4A and 4B). The moderate increase in CXCL2 protein in NIH fibroblasts must be allocated to the increase in cell numbers observed for the differentiation medium (data not shown). By contrast, only mature 3T3-L1 adipocytes produced significant amounts of CXCL2 upon cytokine stimulation (Fig 4A and 4B).

### Adipocyte differentiation was paralleled by changes in cytokine-induced Erk1/2 phosphorylation

**Fig 5:**
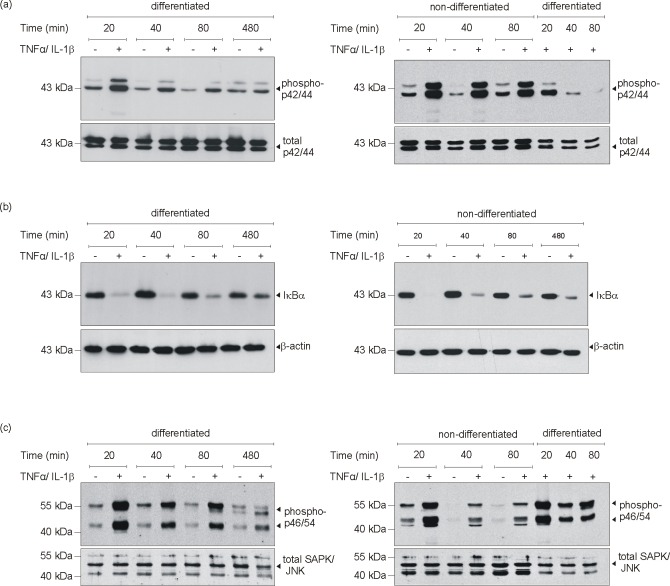
MAPK signaling in cytokine-stimulated differentiated and non-differentiated 3T3-L1 cells. 3T3-L1 pre-adipocytes were differentiated in DMI and DMII for 12 days or remained non-differentiated in basal medium I. Differentiated and non-differentiated 3T3-L1 cells were stimulated with cytokines (25 ng/ml IL-1β, 50 ng/ml TNFα) as indicated. Cell lysates were subsequently analyzed by immunoblot for the presence of phosphorylated p42/44 MAPK (a), degradation of IκBα (b) and phosphorylation of SAPK/JNK (c) as indicated. Total p42/44 (a, lower panel), β-actin (b, lower panel) or total SAPK/JNK (c, lower panel) served as loading controls.

As the cytokines TNFα and IL-1β induced the expression of CXCL2 restrictively upon 3T3-L1 differentiation, we next assessed possible changes in MAPK, NF-κB and stress-activated protein kinase (SAPK)/Jun amino-terminal kinase (JNK) activation upon 3T3-L1 maturation. We did so, as MAPK activation plays a pivotal role in 3T3-L1 differentiation [52,53] and as NF-κB and SAPK/JNK might contribute to the inflammatory response of mature adipocytes (Fig 3D and 3E). As shown in Fig 5A, only non-differentiated 3T3-L1 cells in the fibroblast-like pre-adipocyte state showed a pronounced activation of p42/44 MAPK upon cytokine stimulation. As shown in the left panel (Fig 5A), p42/44 MAPK activation was only weak and short-lived (about 20 min) in differentiated cells. This finding is again visible in the right panel (Fig 5A): cytokines trigger a pronounced and sustained phosphorylation of p44/44 MAPK in non-differentiated 3T3-L1 cells, as again this activation was only very transient and rapidly gone in differentiated cells. Thus, the cytokine-induced sustained p42/44 MAPK activation was markedly attenuated upon differentiation. However, activation of the pro-inflammatory transcription factor NF-κB was not dependent on the maturation state of 3T3-L1 cells (Fig 5B). Interestingly, only SAPK/JNK revealed an increased activation, showing a prolonged and stronger phosphorylation in mature cells following cytokine stimulation (Fig 5C).

### Expression of transcription factors driving adipocyte maturation

**Fig 6:**
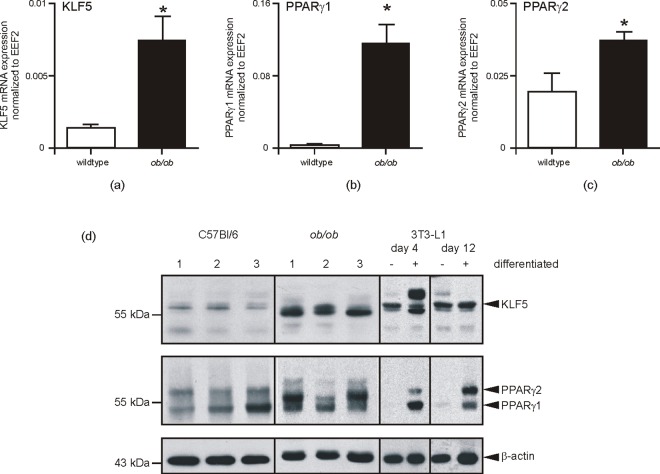
Key transcription factors of adipogenesis are increased in subcutaneous fat of obese mice. qRT-PCR quantification of KLF5 (a), PPARγ1 (b) and PPARγ2 (c) mRNA expression in subcutaneous fat tissue of wildtype and obese (*ob/ob*) mice as indicated. *, p < 0.05 (Student’s unpaired *t* test) as compared to wildtype mice. Bars indicate the mean ± S.D. obtained from fat tissue isolated from four individual animals (n = 4). Subcutaneous fat was analyzed by immunoblot for the presence of KLF5, PPARγ1 and PPARγ2 protein as indicated (d). Lysates from differentiated 3T3-L1 adipocytes served as a positive control for KLF5 and PPAR-specific immunoblot signals. β-actin was used to control loading.

**Fig 7:**
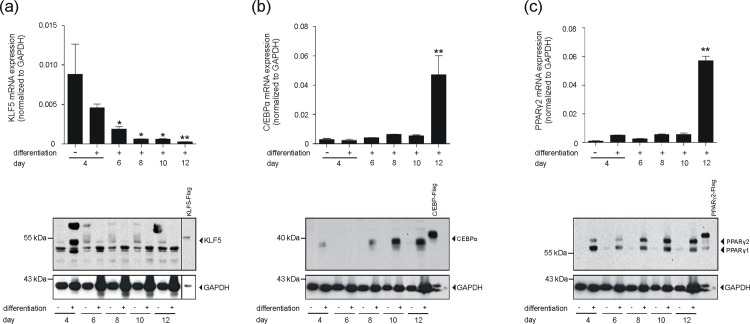
Expression of adipogenic transcription factors in 3T3-L1 cells. 3T3-L1 pre-adipocytes were differentiated in DMI and DMII for 12 days. KLF5 (a), C/EBPα (b) and PPARγ2 (c) mRNA (upper panels) and protein (lower panels) in 3T3-L1 cells was analyzed by qRT-PCR (upper panels) or immunoblot (lower panels) at the indicated time points of differentiation. *, p < 0.05; **, p < 0.01; (Student’s unpaired *t* test) compared to non-differentiated cells. Bars indicate the mean ± S.D. obtained from four independent experiments (n = 4). Recombinant Flag-tagged KLF5 (a), C/EBPα (b) and PPARγ2 (c) served as positive controls in the immunoblots. GAPDH was used to control loading.

Expression of KLF5, C/EBP and PPARγ is a prerequisite for adipocyte differentiation [32–35]. In line with these findings, Fig 6 demonstrates significantly elevated transcription levels of KLF5 (Fig 6A), PPARγ1 (Fig 6B) and PPARγ2 (Fig 6C) in the hypertrophic subcutaneous adipose tissue of obese mice. The respective transcripts appeared to be subsequently translated into protein within the expanded fat tissue in the animals (Fig 6D). A subsequent 3T3-L1 differentiation experiment again strongly related the differential presence of these transcription factors to adipocyte maturation. As shown in Fig 7A, the strong down-regulation of KLF5, expressed in non-differentiated fibroblast-like 3T3-L1 cells, was associated with a proceeding maturation process. By contrast, C/EBPα (Fig 7B) and PPARγ2 (Fig 7C) were absent in fibroblastic 3T3-L1 pre-adipocytes, but were markedly up-regulated during late adipocyte differentiation.

### Dependence of PPARγ transcription factor expression for adipocyte maturation provides a target to reduce pro-inflammatory properties of mature adipocytes

**Fig 8:**
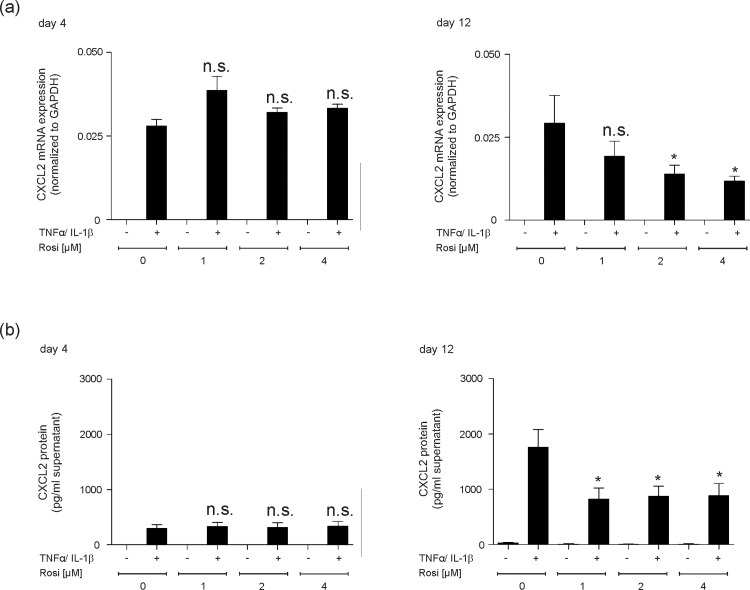
PPARγ-agonist rosiglitazone reduces CXCL2 expression in finally differentiated 3T3-L1 adipocytes. 3T3-L1 pre-adipocytes were differentiated in differentiation medium for 4 days or 12 days as indicated. Differentiated 3T3-L1 cells were then stimulated with cytokines (25 ng/ml IL-1β, 50 ng/ml TNFα) for 8 h in the presence of increasing concentrations of rosiglitazone (1–4 μM). CXCL2 mRNA expression was analyzed by qRT-PCR (a). CXCL2 protein release into cell culture supernatants was determined by ELISA (b). *, p < 0.05; n.s., not significant (Student’s unpaired *t* test) compared to cytokine-treated, but rosiglitazone-free cells. Bars indicate the mean ± S.D. obtained from four independent experiments (n = 4).

**Fig 9:**
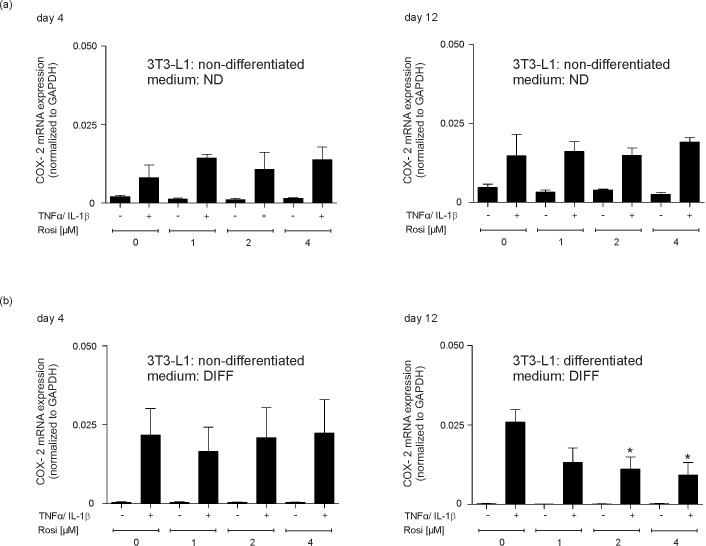
PPARγ-agonist rosiglitazone reduces Cox-2 expression in finally differentiated 3T3-L1 adipocytes. 3T3-L1 pre-adipocytes remained non-differentiated in non-differentiating control medium (ND) or were stimulated to induce the differentiation process in differentiating medium (*DIFF*) for 4 days or 12 days as indicated. 3T3-L1 cells in non-differentiating medium (ND) (a) or differentiating medium (*DIFF*) (b) were then stimulated with cytokines (25 ng/ml IL-1β, 50 ng/ml TNFα) for 8 h in the presence of increasing concentrations of rosiglitazone (1–4 μM) before (day 4) and after (day 12) maturation to adipocytes. Cox-2 mRNA expression was analyzed by qRT-PCR. *, p < 0.05; n.s., not significant (Student’s unpaired *t* test) compared to cytokine-treated, but rosiglitazone-free cells. Bars indicate the mean ± S.D. obtained from four independent experiments (n = 4).

One major role of PPARγ is the mediation of anti-inflammatory responses [54]. As this particular transcription factor is an essential prerequisite for final adipocyte maturation [34,35] and strongly expressed upon 3T3-L1 final differentiation (Fig 7C), it was tempting to argue that agonistic activation of PPARγ might serve to reduce cytokine-triggered inflammatory responses (Figs 3 and 4) in mature adipocytes. In line with this notion, the absence of PPARγ in non-differentiated 3T3-L1 cells (Fig 7C) might provide the explanation for the failure of rosiglitazone to reduce CXCL2 (S2 Fig) and CCL5 (S2 Fig) expression at early and late time points of cytokine stimulation in the cells. In clear contrast to conditions in non-differentiated 3T3-L1 pre-adipocytes, we again discovered a cytokine-inducible expression of CXCL2 in differentiating 3T3-L1 cells (Fig 8). As shown in detail, the cytokine-induced CXCL2 expression could not be suppressed by the PPARγ agonist rosiglitazone (Fig 8A and 8B, left panel), as this particular transcription factor is not expressed at that pre-mature stage of differentiation (Fig 7C). However, finally differentiated 3T3-L1 adipocytes were now capable to respond to increasing levels of rosiglitazone, as PPARγ was expressed in the cells during late differentiation (Fig 7C). As shown in Fig 8A and 8B (right panel), rosiglitazone indeed triggered a significant reduction of CXCL2 expression and release from mature 3T3-L1 adipocytes. To strengthen our observation of anti-inflammatory effects of PPARγ agonists in differentiated 3T3-L1 adipocytes, we included the analysis of the pro-inflammatory enzyme Cox-2. In accordance to CXCL2 (Fig 8) and CCL5 (S3 Fig) expression and release, Cox-2 expression was induced by cytokines in early and late adipocyte maturation stages (Fig 9). Here, it is again important to notice that 3T3-L1 cells were still in a non-differentiated state upon the early 4 day-treatment time point in differentiating medium (Fig 9A and 9B, left panels). Again, the response to rosiglitazone was restricted to mature 3T3-L1 adipocytes, which showed a marked expressional reduction of the pro-inflammatory Cox-2 enzyme (Fig 9).

**Fig 10:**
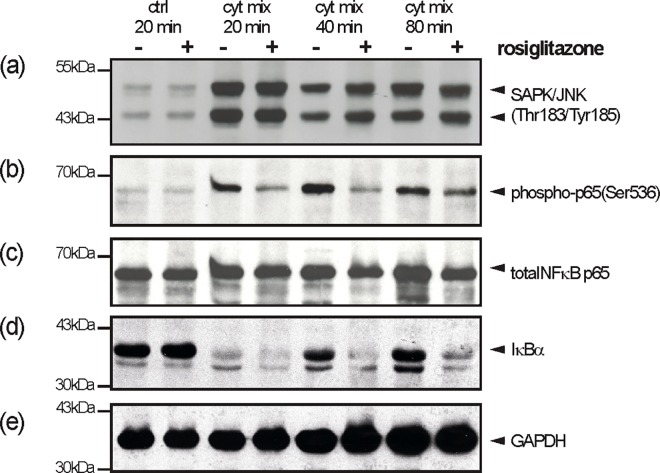
Effects of rosiglitazone on SAPK/JNK and NF-κB signaling in differentiated 3T3-L1 cells. 3T3-L1 pre-adipocytes were differentiated in DMI and DMII for 12 days. Differentiated 3T3-L1 cells were stimulated with cytokines (25 ng/ml IL-1β, 50 ng/ml TNFα) in the presence or absence of rosiglitazone (4μM) as indicated. Cell lysates were subsequently analyzed by immunoblot for the presence of phosphorylated SAPK/JNK (a), the phosphorylation of the NF-κb p65 subunit (b) and degradation of IκBα (d). Total NF-κb p65 (c) and GAPDH (e) served as loading controls.

As we had shown an activation of the NF-κB (via degradation of IκBα) and SAPK/JNK signalling pathways by cytokines in differentiated 3T3-L1 cells (Fig 5B and 5C), we were interested for the impact of rosiglitazone on cytokine-induced NF-κB and SAPK/JNK signalling. The CXCL2 promoter contains a conserved NF-κB consensus motif that is pivotal to transcription of the CXCL2 gene [55], suggesting an interference with the NF-κB pathway as a potential mode of action in rosiglitazone-mediated attenuation of CXCL2 expression (Fig 8). As shown in Fig 10A, rosiglitazone did not interfere with the activation of SAPK/JNK at all. By contrast, it is interesting to note that rosiglitazone markedly suppressed the re-appearance of degraded IκBα in the presence of cytokines (Fig 10D). However, the prolonged reduction in IκBα protein did not translate into an increased expression of CXCL2 (Fig 8), as rosiglitazone markedly suppressed the phosphorylation-based (Ser536) activation of the NF-κB p65 subunit (Fig 10B).

### Rosiglitazone treatment improves disturbed wounds in obese mice

**Fig 11:**
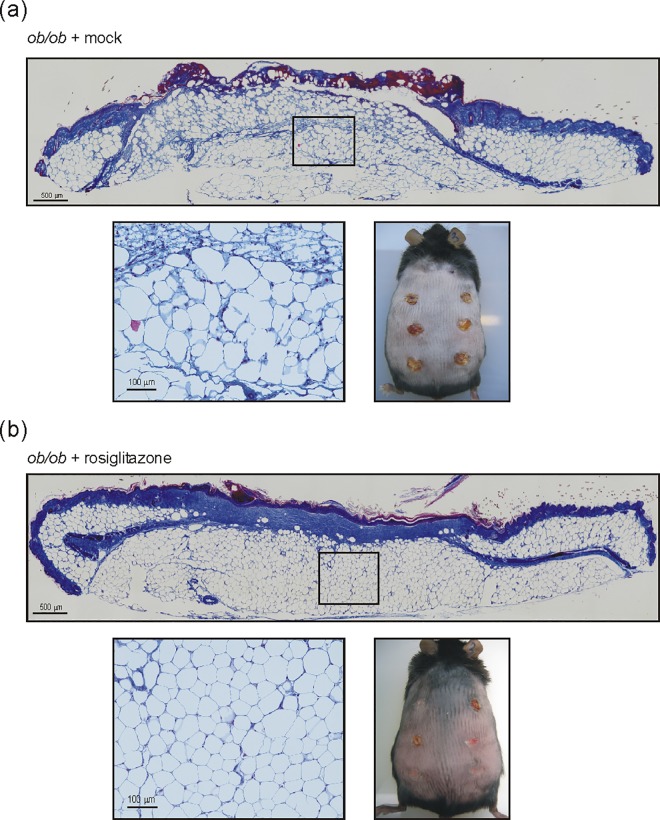
Rosiglitazone improves wound healing and subcutaneous fat cell morphology. Formalin-fixed and paraffin-embedded 13-day wound sections of mock- (a) or rosiglitazone-(b) treated mice (0.5 mg/kg/day) were assessed by AZAN trichrome staining. Collagen deposition is indicated by the blue color. The squares indicate the localizations of the respective magnifications. Scale bars are given in the photographs. Appearance of back wounds upon a 13-day mock (a) or rosiglitazone (b) administration in *ob/ob* mice is shown in the lower right panels.

Wound tissues in diabetic and obese mice are severely disturbed and particularly characterized by an augmented and prolonged inflammatory response [17,20]. However, a contribution of the expanded subcutaneous adipose tissue mass to wound inflammatory responses has not yet been analyzed. We had assessed significantly elevated levels of PPARγ isoforms in subcutaneous adipose tissue in obese mice (Fig 6B and 6C). Therefore, we treated diabetic and obese *ob/ob* mice using the PPARγ agonist rosiglitazone starting 2 days prior to wounding. To verify the known insulin-sensitizing effect of thiazolidinediones, we assessed the effects of rosiglitazone treatment on body weight and fasting or challenged blood glucose levels in the mice. As given in S4 Fig, we observed a significant increase in body weight upon rosiglitazone treatment (S4A Fig), which is a well-known side effect of PPARγ agonists [40]. In line, the insulin-sensitizing effect of rosiglitazone was reflected in reduced fasting blood glucose levels (S4B Fig) and an improved insulin sensitivity after oral glucose administration (S4C Fig). In parallel, rosiglitazone markedly improved the disturbed wound conditions in the animals (Fig 11). Wound tissue from rosiglitazone-treated mice showed a complete re-epithelialization and formation of a collagen-enriched neo-dermis (Fig 11B, upper panel). Mock-treated *ob/ob* mice revealed still impaired wounds with atrophied epithelia and the failure to form a collagen-rich granulation tissue (Fig 11A, upper panel). The rosiglitazone-mediated improvement in wound morphology is again reflected in the overall wound appearance: most wounds of rosiglitazone-treated mice have been visibly re-epithelialized (Fig 11B, lower right panel), whereas wounds from mock-treated animals remained still covered by a robust scab (Fig 11A, lower right panel). Here it is important to note that the PPARγ agonist markedly changed the morphology of the adipose tissue underlying the wound areas. Whereas mock-treated mice show an irregular organization of mostly enlarged adipocytes (Fig 11A, lower left panel), rosiglitazone treatment led to a very regular pattern of adipocytes of similar size (Fig 11B, lower left panel), arguing for an effect of the drug on adipocytes.

**Fig 12:**
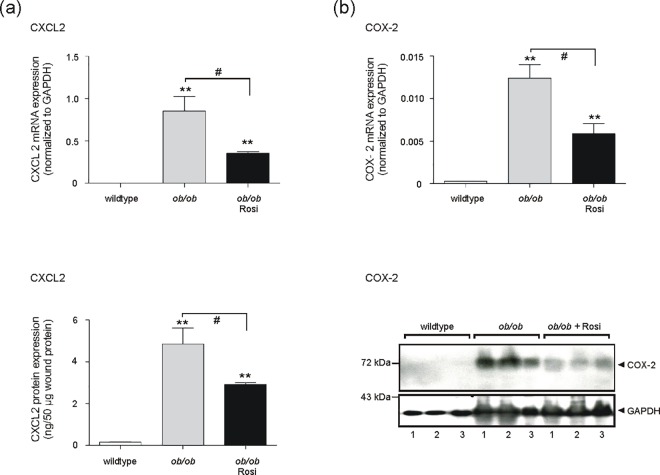
Rosiglitzone attenuates exacerbated CXCL2 and Cox-2 expression in wounds of obese mice. Quantification of CXCL2 (a) and Cox-2 (b) expression in 13-day wound tissue of wildtype and obese *ob/ob* mice as indicated. mRNA expression was assessed by qRT-PCR, protein expression was analyzed by ELISA for CXCL2, or by immunoblot for Cox-2. GAPDH was used to control loading. **, p < 0.01; (Student’s unpaired *t* test) as compared to wildtype mice. #, p < 0.05 (Student’s unpaired *t*-test) as indicated by the brackets. Bars indicate the mean ± S.D. obtained from wound tissue (three wounds per animal) isolated from four individual animals (n = 4).

Finally, we checked the wound tissues of mock- and rosiglitazone-treated obese mice for the expression CXCL2 and Cox-2. Both genes could be induced by cytokines and inhibited by rosiglitazone in mature 3T3-L1 adipocytes (Figs 8B and 9B). Notably, rosiglitazone-improved wound tissue and adipocyte morphology (Fig 11) was paralleled by a marked reduction in both CXCL2 (Fig 12A) and Cox-2 (Fig 12B) mRNA and protein expression in skin wounds.

## Discussion

The strong demand towards an essential therapeutic improvement in the treatment of diabetes-associated ulcerations has not been yet resolved satisfactorily. In line, a most recent population-based cohort study from the United Kingdom published in 2015, particularly indicated the diabetic foot ulcer as a major warning sign for mortality. From patients that developed diabetic foot ulcers, about 42% died within 5 years [56]. These currently published numbers point out that a long period of research was not sufficient to significantly improve ulcer treatment strategies, as comparably low survival rates of diabetic foot ulcer patients had been published about 15 years ago [5,6]. Probably, the clinical situation will grow even more acute with the expected rise of diabetes and obesity in the next decades [2–4]. Although conditions of human diabetic ulceration [57] only partially match with the disturbed wound healing in obese and diabetic mice, there are no better experimental alternatives for the use of both mouse models in diabetes-associated wound healing research. Remarkably, the notion that diabetic *ob/ob* and *db/db* mice as well suffer from obesity and thus a massive subcutaneous adipose tissue mass has been ignored in most studies on cutaneous wound regeneration in the animals. Thus, these studies did not assess the potential contribution of the expanded subcutaneous adipose tissue to impaired wound healing conditions [12–15,17,20,58]. However, histologic analyses of wounds from obese and diabetic mice (please refer to Fig 10) clearly show the dominant presence of a large mass of subcutaneous adipocytes underlying the wound sites. Taking this into consideration, it is all the more surprising that the molecular and cellular response of subcutaneous adipocytes to injury have not been analyzed in detail yet.

We found an upward trend for FABP4 mRNA and an increased expression of leptin mRNA in subcutaneous adipose tissue from *ob/ob*-mice. This finding is in good accordance with the established knowledge of increased FABP4 [59,60] and leptin [61] levels under conditions of obesity in mice. In addition, the presence of elevated expression levels of KLF5 as well as PPARγ in subcutaneous adipocytes argues for increased numbers of fully differentiated mature adipocytes associated with conditions of obesity, as both transcription factors are known to contribute to the sequential progression of adipocyte maturation [32, 34–36]. We compared the observed elevated expression of CXCL2, leptin, KLF5 and PPARγ with the comprehensive data sets from an elementary study performed on obese mice [50]. This study had identified 1,304 transcripts that significantly correlated with body mass in C57Bl/6 mice. Interestingly, this important study showed that the average adipocyte cross-sectional area was a strong predictor of the percentage of macrophages within the fat depots. The authors state that macrophages located in between the hypertrophic adipocytes are most likely to account for the expressional changes of adipose tissue in obesity. Here, the 100 adipose tissue transcripts whose expression most significantly correlated with body mass were preferentially expressed in macrophages. Interestingly, these particular macrophage-derived transcripts did not contain CXCL2, CCL5, leptin, FABP4, KLF5 or PPARγ. Therefore, it is tempting to hypothesize here that not adipose tissue macrophages but likely the adipocytes itself might also represent a cellular source of the mentioned transcripts. Moreover, the concurrent presence of KLF5, expressed during early stages of adipocyte differentiation [32], and PPARγ, a master regulator of late adipocyte maturation [34–36], suggests a concurrent presence of premature and mature adipocytes in wound fat tissue. However, subcutaneous fat from wound margin areas and non-wounded skin exhibit the overall presence of large adipocytes. These cells are characterized by a small cytoplasmic margin area and a large central vacuole for fat storage. Therefore, it is reasonable to suggest that subcutaneous fat from non-wounded skin mostly consists of mature adipocytes

In addition, induction of gene expression by external signals often require activation of intracellular kinases. In particular, the p42/44 and p38 MAPK represent pivotal regulators of adipocyte differentiation [62]. In our experimental setup, we used the cytokines TNFα and IL-1β to induce an inflammatory response from premature and differentiated 3T3L1-adipocytes. This treatment therefore might affect the interplay of adipocyte differentiation and the release of inflammatory mediators from the cells. It has to be considered that a cytokine-treatment of cells to induce the release of CXCL2 might also directly influence the cellular differentiation process through activation of MAPKs. In particular, p44/44 MAPK appears to be pivotal to adipocyte differentiation, although its role has not been conclusively characterized. *In vitro* studies on 3T3L1-adipocytes have shown that down-regulation of p42/44 MAPK activity, particularly mediated by an increased MAPK-phosphatase-1 activity antagonized the differentiation process [52,63]. In sharp contrast, additional studies implicate p42/44 MAPK activation as a key element driving adipocyte maturation in vitro [64] and in mice [62]. However, we observed a much stronger activation of p42/44 MAPK upon cytokine stimulation in non-differentiated 3T3L1-adipocytes. This finding argues towards the described reduction of MAPK activation during adipocyte differentiation. However, this notion also implies that p42/44 MAPK did not actually contribute to the inflammatory response of mature 3T3-adipocytes. As p42/44 MAPK activation most likely appeared to be not involved in the inflammatory response of differentiated adipocytes, we also assessed the activation of the classic pro-inflammatory transcription factor nuclear factor (NF)-κB in the cells [65]. NFκB protein must be released from its inhibitory regulator, the inhibitor of κB (IκB), to exert NFκB transcriptional activity [65]. Interestingly, activation of the classic pro-inflammatory NFκB pathway was not altered upon adipocyte maturation. The augmented and prolonged degradation of the inhibitory IκBα protein in wound tissue from *ob/ob* mice suggests that maturation of adipocytes in the subcutaneous fat compartment did not contribute to the observed exacerbation of NFκB signaling in obese mice [12]. However, rosiglitazone was capable to reduce CXCL2 expression in wound tissue of ob/ob mice. Transcription of murine CXCL2 is dependent on a conserved NF-κB consensus motif 51 to 70 bp 5’ from the transcription start site [55]. Thus, it is tempting to argue here that the attenuated phosphorylation of the p65 NF-κB subunit observed for rosiglitazone in differentiated 3T3-L1 cells *in vitro* might also contribute to the decreased expression of CXCL2 upon rosiglitazone treatment in *ob/ob* mice. There is no evidence for a role of JNK in adipocyte differentiation, although it is clear that JNK1 decreases insulin sensitivity of the cells by phosphorylation of insulin receptor substrate (IRS)-1 [66]. However, the increased activation of JNK1/2 in mature adipocytes, as defined in this study, might be a marker of the elevated inflammatory response of the cells, as JNK1/2 regulates inflammatory processes and is associated with a variety of inflammatory diseases [67].

It is now established that immune responses and obesity are tightly connected and that insulin resistance has to be regarded as a functional consequence of adipose tissue-driven inflammatory processes [68]. Interestingly, our previous data have shown that also normal skin and wound tissue in obese mice was characterized by a distinct insulin resistance that contributed to wound healing disorders in the animals [58]. Thiazolidinediones (TZD), such as rosiglitazone, represent a class of antidiabetic drugs that are capable of improving insulin resistance [69] through activation of the nuclear hormone receptor PPARγ [37], which serves also as a master regulator of late adipocyte maturation [34–36]. Thus, it becomes obvious here that PPARγ exert regulatory functions at the crossroads between insulin resistance, inflammation and adipocyte differentiation. This is all the more important, as insulin resistance is a consequence of the release of inflammatory mediators from growing adipose tissue under conditions of obesity [68,70]. In line with this notion, TZDs interfere with this inflammatory signaling process of adipocytes to muscle and liver and improve insulin sensitivity through down-regulation of TNFα and IL-6 in adipose tissue [71]. Furthermore, this finding clearly corresponds with the potency of rosiglitazone to suppress the expression of CXCL2 and Cox-2 restrictively in differentiated adipocytes. CXCL2 and Cox-2 represent pro-inflammatory wound proteins and are associated with a prolonged wound inflammation in obesity-impaired wound healing conditions [17,20]. Activation of PPARγ by rosiglitazone, as expressed in mature adipocytes, improved the morphology of subcutaneous adipocytes and markedly reduced the expression of the pro-inflammatory proteins CXCL2 and Cox-2 in our experimental setting.

Moreover, these rosiglitazone-induced conditions were paralleled by a significant improvement of wound closure and new tissue formation in obese mice. Here it is tempting to argue in favor of the well-known insulin-sensitizing effects of thiazolidinedones [40], which might preferentially account for the improved tissue repair in *ob/ob* mice rather than the attenuated inflammatory conditions at the wound site. However, this notion actually does not reflect the actual conditions at impaired wound sites. The attenuation of wound inflammatory conditions in *ob/ob* mice by antibody-mediated depletion of wound macrophages markedly restored a normal wound healing in disturbed wounds in the full presence of unaltered hyperglycemic and hyperinsulinemic conditions in the animals [25]. These findings strongly suggest that indeed the control of wound inflammation, but not an insulin-sensitizing effect, represents a prerequisite for an improved repair in obese mice. Therefore, our data implicate that subcutaneous adipocytes might also contribute to diabetes-associated wound healing disorders in obese mice. However, as PPARγ2 is a specific transcription factor in mature adipocytes, this transcription factor provides an effective therapeutical target to reduce the production of inflammatory mediators from adipocytes and improve disturbed tissue regeneration in conditions of obesity.

## Supporting Information

S1 Fig Skin morphology demonstrating isolated subcutaneous adipose tissue layers. Formalin-fixed and paraffin-embedded sections from non-wounded skin of wildtype (upper panels) and ob/ob mice (lower panel) were assessed by AZAN trichrome staining to show the isolated subcutaneous adipose tissue compartment. Scale bars are given in the photographs.
(TIF)

S2 Fig CXCL2 is not induced in non-differentiated 3T3-L1 cells. 3T3-L1 pre-adipocytes were cultured in normal control medium (DMEM) for 4 days or 12 days as indicated. Non-differentiated 3T3-L1 cells were then stimulated with cytokines (25 ng/ml IL-1β, 50 ng/ml TNFα) for 8h in the presence of increasing concentrations of rosiglitazone (1–4 μM). CXCL2 mRNA expression was analyzed by qRT-PCR (a). CXCL2 protein release into cell culture supernatants was determined by ELISA (b). n.s., not significant (Student’s unpaired *t* test) compared to cytokine-treated, but rosiglitazone-free cells. Bars indicate the mean ± S.D. obtained from four independent experiments (n = 4).
(TIF)

S3 Fig Rosiglitazone suppresses cytokine-induced CCL5 mRNA expression only in mature 3T3-L1 adipocytes. 3T3-L1 pre-adipocytes remained undifferentiated in control medium (DMEM) (a) or were differentiated in differentiation medium (b) for 4 days or 12 days as indicated. At the indicated time points, non-differentiated (a) and differentiating (b) 3T3-L1 cells were stimulated with cytokines (25 ng/ml IL-1ββ, 50 ng/ml TNFα) for 8h in the presence of increasing concentrations of rosiglitazone (1–4 μM). CCL5 mRNA expression was then analyzed by qRT-PCR. **, p < 0.01; *, p < 0.05; (Student’s unpaired *t* test) as indicated by the brackets. Bars indicate the mean ± S.D. obtained from four independent experiments (n = 4).
(TIF)

S4 Fig Effects of oral rosiglitazone treatment on body weight and insulin sensitivity in *ob/ob* mice. ob/ob mice were treated with rosiglitazone (0.5 mg/kg/day) two days prior to wounding followed by an daily administration during healing until day 13 post-wounding. At day 13 post-wounding, animals were assessed for body weight (a) and fasting blood glucose (b). Glucose tolerance was assessed by determination of blood glucose levels following oral administration of glucose (1.5 g/kg body weight) for 120 min (c).
(TIF)
