# Supplementary figures and images for: Anti-Inflammatory Effects of Rosiglitazone in Obesity-Impaired Wound Healing Depend on Adipocyte Differentiation
Source: PLoS One. 2016 Dec 19;11(12):e0168562. doi: 10.1371/journal.pone.0168562 (PMC5167406; doi:10.1371/journal.pone.0168562)

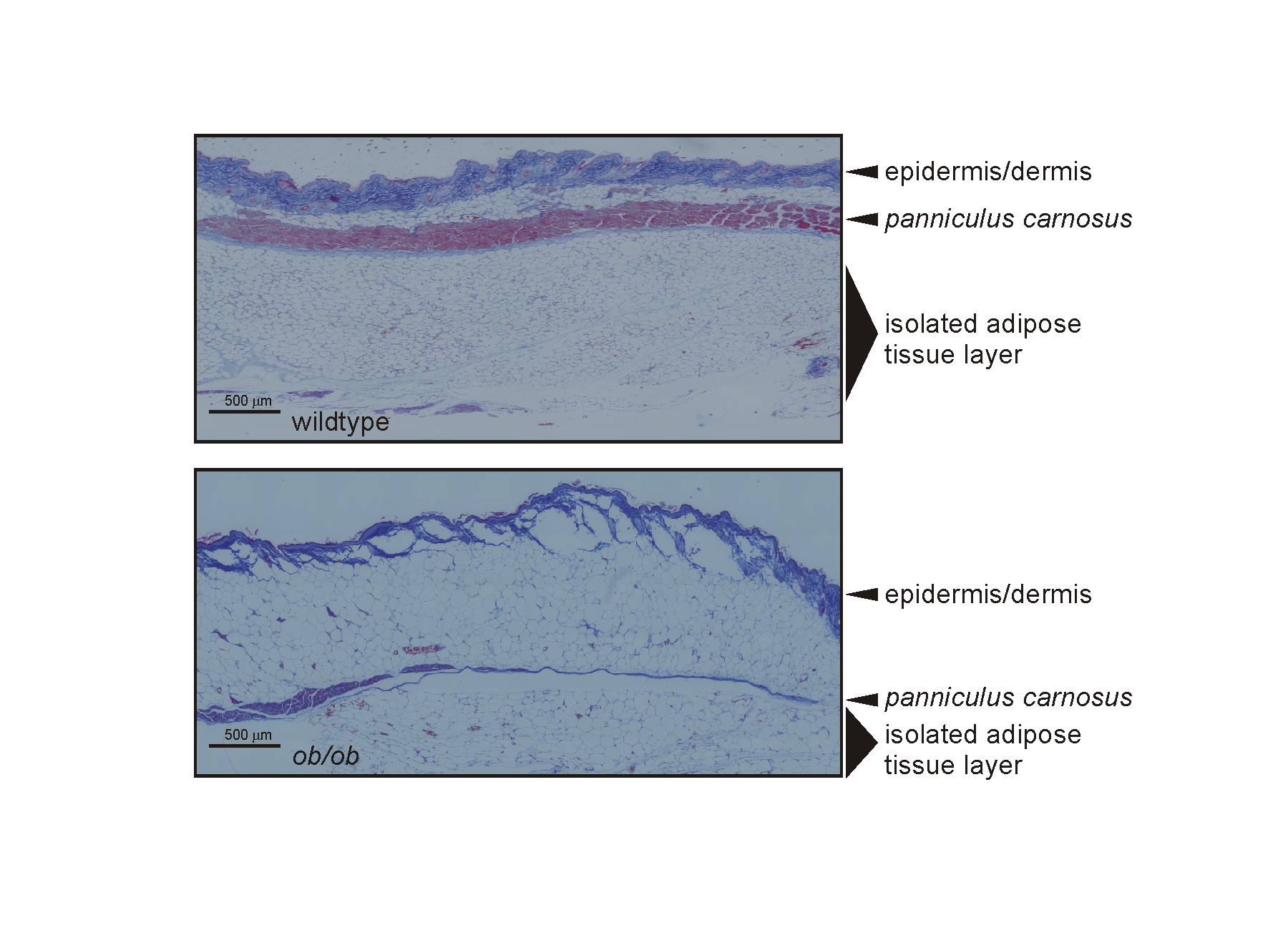

Supplement: S1 Fig — Formalin-fixed and paraffin-embedded sections from non-wounded skin of wildtype (upper panels) and ob/ob mice (lower panel) were assessed by AZAN trichrome staining to show the isolated subcutaneous adipose tissue compartment. Scale bars are given in the photographs. (TIF) [file pone.0168562.s001.TIF]

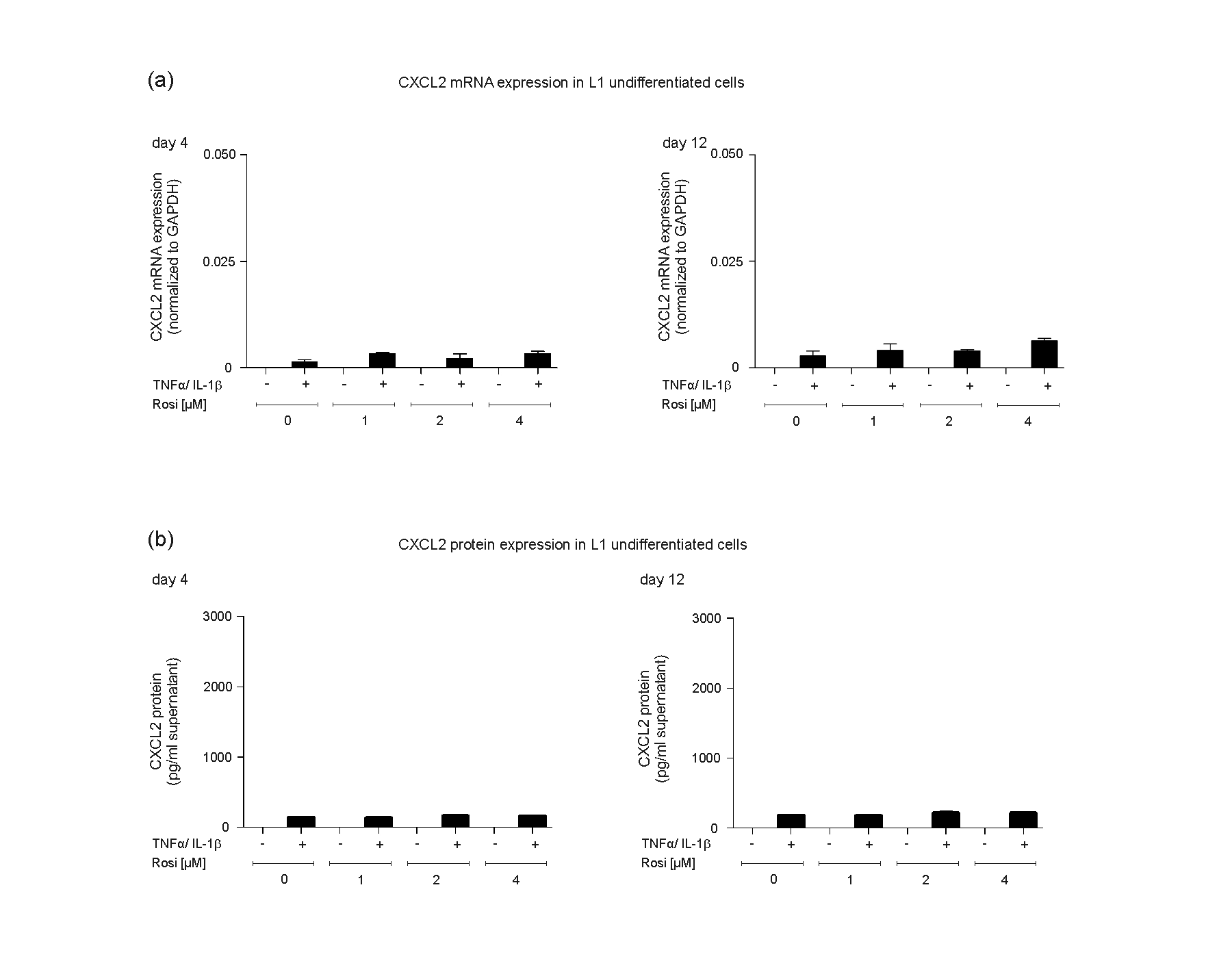

Supplement: S2 Fig — 3T3-L1 pre-adipocytes were cultured in normal control medium (DMEM) for 4 days or 12 days as indicated. Non-differentiated 3T3-L1 cells were then stimulated with cytokines (25 ng/ml IL-1β, 50 ng/ml TNFα) for 8h in the presence of increasing concentrations of rosiglitazone (1–4 μM). CXCL2 mRNA expression was analyzed by qRT-PCR (a). CXCL2 protein release into cell culture supernatants was determined by ELISA (b). n.s., not significant (Student’s unpaired t test) compared to cytokine-treated, but rosiglitazone-free cells. Bars indicate the mean ± S.D. obtained from four independent experiments (n = 4). (TIF) [file pone.0168562.s002.TIF]

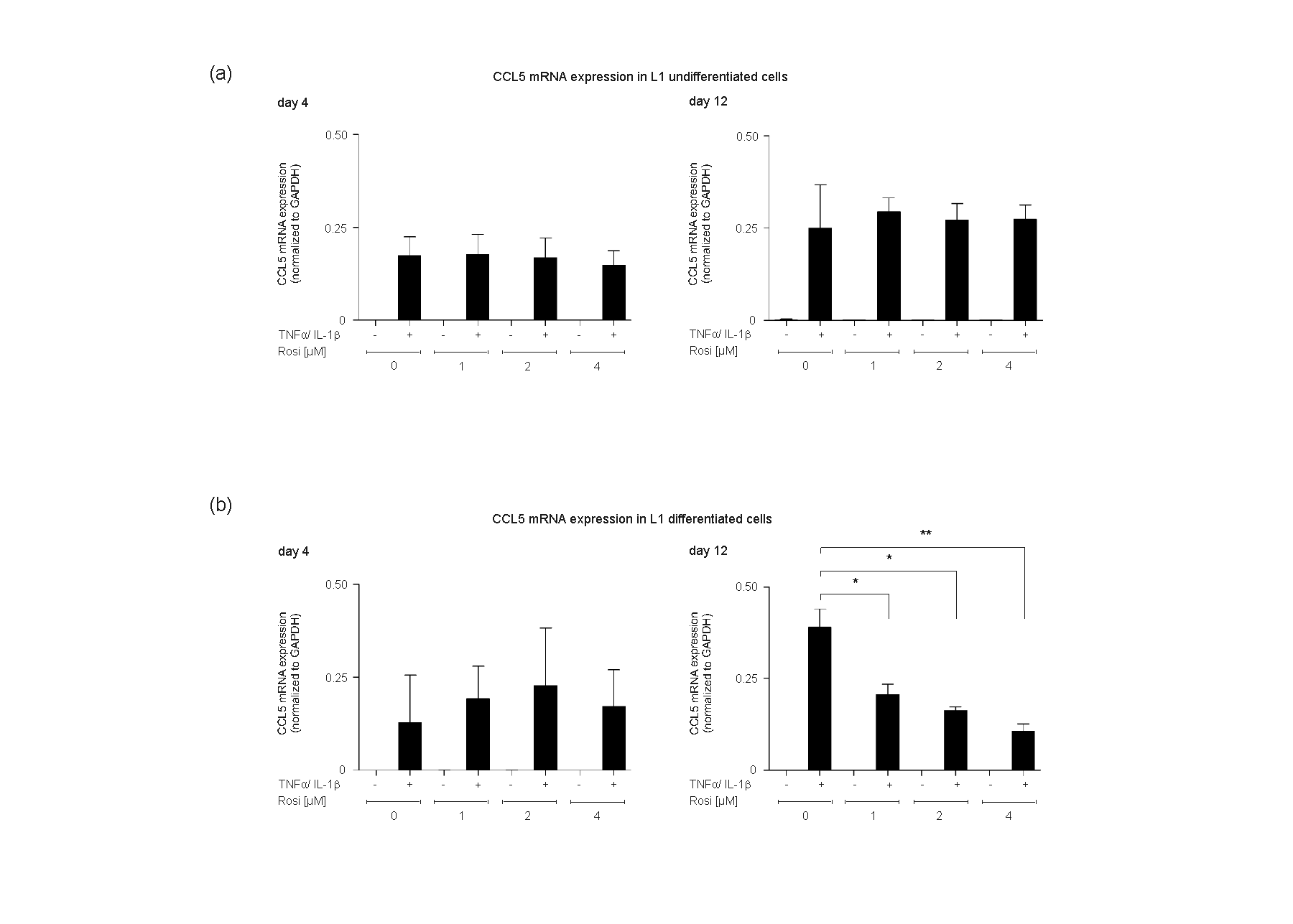

Supplement: S3 Fig — 3T3-L1 pre-adipocytes remained undifferentiated in control medium (DMEM) (a) or were differentiated in differentiation medium (b) for 4 days or 12 days as indicated. At the indicated time points, non-differentiated (a) and differentiating (b) 3T3-L1 cells were stimulated with cytokines (25 ng/ml IL-1ββ, 50 ng/ml TNFα) for 8h in the presence of increasing concentrations of rosiglitazone (1–4 μM). CCL5 mRNA expression was then analyzed by qRT-PCR. **, p < 0.01; *, p < 0.05; (Student’s unpaired t test) as indicated by the brackets. Bars indicate the mean ± S.D. obtained from four independent experiments (n = 4). (TIF) [file pone.0168562.s003.TIF]

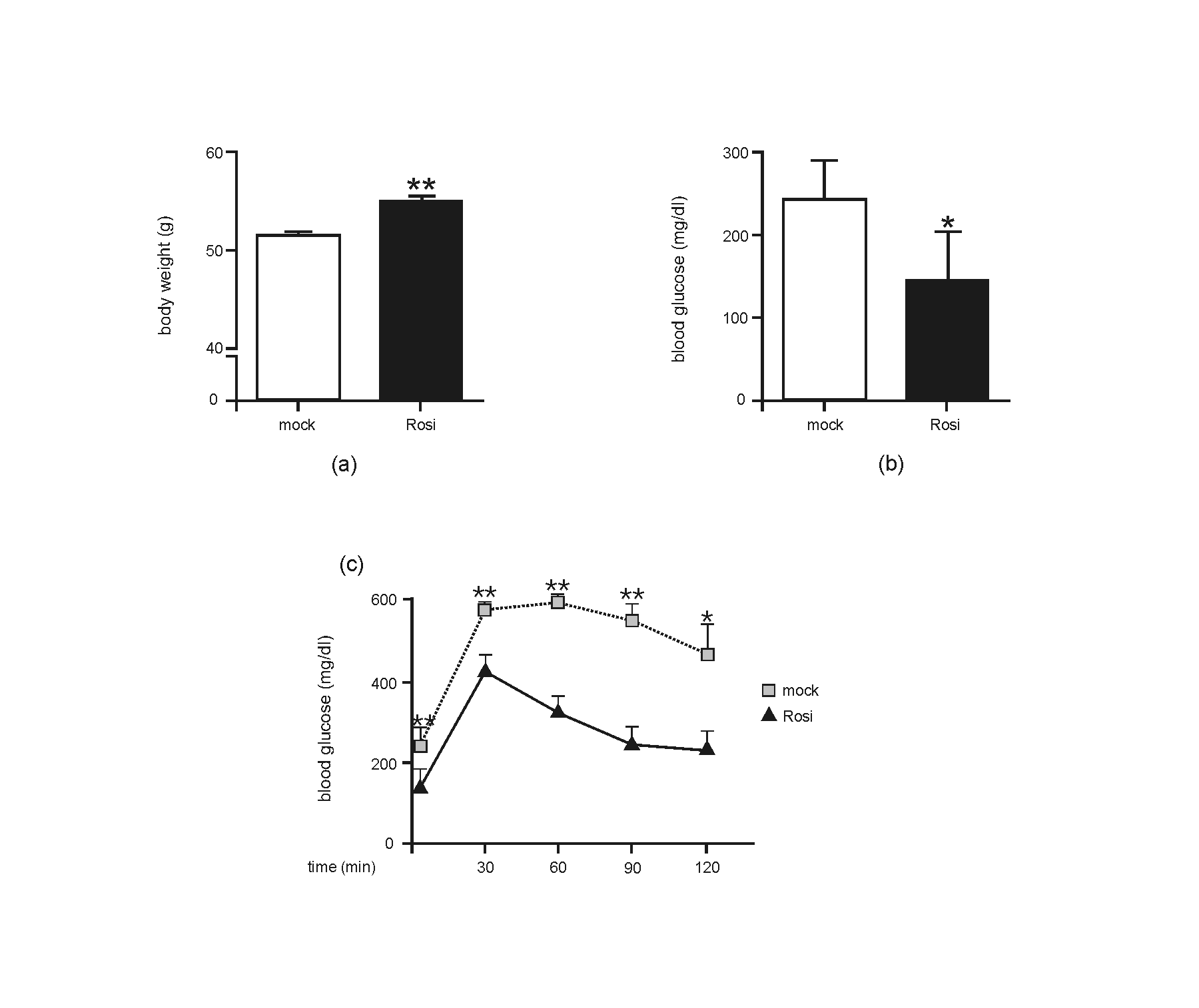

Supplement: S4 Fig — ob/ob mice were treated with rosiglitazone (0.5 mg/kg/day) two days prior to wounding followed by an daily administration during healing until day 13 post-wounding. At day 13 post-wounding, animals were assessed for body weight (a) and fasting blood glucose (b). Glucose tolerance was assessed by determination of blood glucose levels following oral administration of glucose (1.5 g/kg body weight) for 120 min (c). (TIF) [file pone.0168562.s004.TIF]
